# Labor Epidural Analgesia and Postpartum Depression

**DOI:** 10.17352/2455-5460.000014

**Published:** 2016-11-14

**Authors:** Catherine D Tobin, Sylvia H Wilson, Latha Hebbar, Laura L Roberts, Bethany J Wolf, Constance Guille

**Affiliations:** Medical University of South Carolina, USA

## Abstract

**Introduction:**

Epidural labor analgesia may decrease the risk of postpartum depression (PPD).

**Methods:**

In a secondary analysis of a prospective study, the association between epidural utilization and PPD was evaluated using a Fisher’s exact test. PPD was defined as an Edinburgh Postnatal Depression score of ≥ 10 at 6–8 weeks postpartum.

**Results:**

20% (13/65) of women meet criteria for PDD. 24% (n=12/50) of women who received epidural labor analgesia developed PPD, compared to 6.7% (n=1/15) of women who did not receive epidural labor analgesia (*P* = 0.27).

**Conclusions:**

Labor epidural analgesia did not reduce the risk of postpartum depression.

## Introduction

Postpartum depression (PPD) is a common psychiatric disorder affecting 11–20.4% of childbearing women and is a major public health concern [1]. PPD poses significant risks to mothers and their children, including risk of maternal suicide, infanticide, poor infant growth and development, and decreased maternal–newborn attachment. PPD risk reduction strategies are greatly needed and are an important area of current investigation.

Causes of PPD are characterized as multifactorial. While a number of important risk factors for PPD have been identified including anxiety and depression during pregnancy, lack of social support, previous perinatal loss [2], many of these factors are not modifiable making it difficult to reduce the risk of PPD. However, labor pain is often characterized as severe [3] and has been associated with post-traumatic stress disorder [4]. Similarly, a link between mood disorders in the postpartum period and pain in childbirth has also been noted [5]. Consequently, it is hypothesized that the immense psychological stress stimulated by labor pain may result in further neuropsychological consequences including PPD development. Therefore, analgesia during childbirth may ameliorate this stress response and reduce PPD rates.

Labor epidural analgesia reduces the pain of childbirth. Prior studies have suggested that labor epidural analgesia for childbirth may lower the risk of PPD [6,7]. However, other analgesia methods for labor including nitrous oxide and acupuncture have not been associated with decreased rates of PPD [7]. As epidural labor analgesia is increasingly accessible, this is an exciting area of investigation, but replication of these findings is needed. The aim of this study was to examine the association of labor epidural analgesia and risk of PPD.

## Methods

This study was a secondary analysis of an IRB approved prospective cohort study conducted at the Medical University of South Carolina in Charleston, South Carolina, United States. The original study, yet to be published, examined the association of blood biological markers and peripartum depression. The original study also prospectively evaluated for antepartum depression at 8–10 weeks and 24–28 weeks gestation. Epidural status at time of delivery was noted. Women were screened for PPD on their routine postpartum visit at 6–8 weeks postpartum using the Edinburgh Postnatal Depression Scale (EDPS) score of ≥ 10. EPDS has been described as the most common screening tool for postpartum depression evaluation [1]. The EDPS is a 10-item questionnaire and is a validated screening tool for depression in pregnant and postpartum women [9]. An EPDS score of ≥ 10 has been used in other studies to identify PPD [2,8]. A variety of EPDS cut-off scores ranging from 9 to 13 have been used for diagnosis.

Participant’s corresponding demographic information was gathered antepartum including age, BMI, race, parity, gravida, relationship status with the father, income level, education level and history of psychiatric illness. Following delivery, participant’s charts were reviewed to identify utilization or non-utilization of epidural labor analgesia as well as gestational age of the baby at delivery.

Statistical analyses were performed using Fisher’s exact tests to examine the association between labor epidural and PPD at 6–8 weeks postpartum and other dichotomous variables. T-tests or Wilcoxon rank sum tests were used for continuous variables where appropriate.

## Results

**Figure 1:**
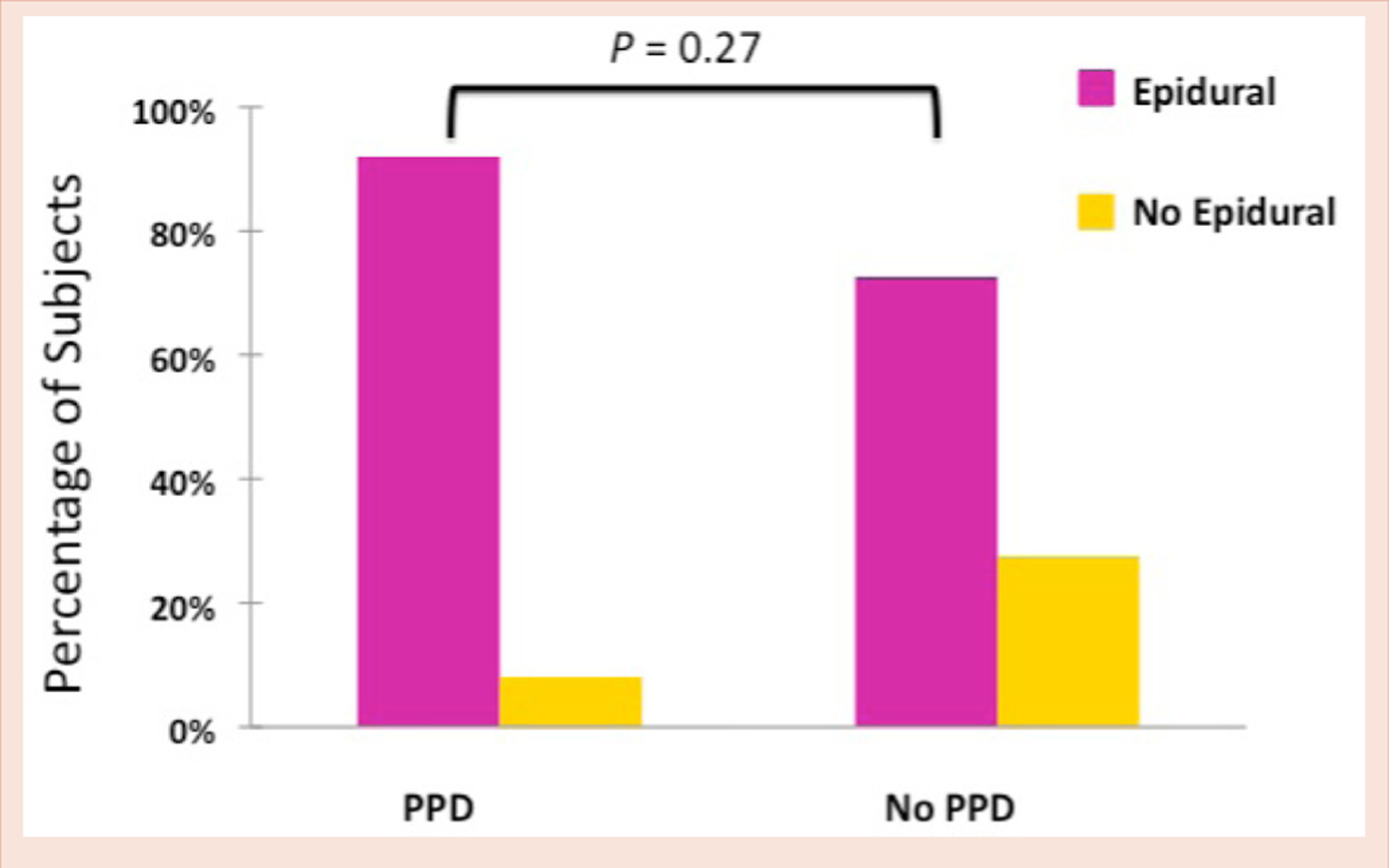
Epidural choice by postpartum depression status.

Epidural utilization during labor, and PPD status were available for 65 parturients. Demographic characteristics of parturients did not differ between subjects with EDPS score of < 10 compared to those with EDPS score ≥ 10 (Table 1). At 6–8 weeks postpartum, 20% (13/65) of women meet criteria for PPD. Epidural labor analgesia was utilized by the majority of parturients (76.9%; 50/65; Table 1). Parturients who received epidural labor analgesia had a 24% (n=12/50) incidence of PPD compared to a rate of 6.7% (n=1/15) among parturients who did not receive labor analgesia (*P* = 0.27; Figure 1).

Women without PPD had an overall lower BMI compared to women with PPD. Obesity was seen in 28.9% of parturients with no PPD, but in 53.9% of parturients with PPD. Occurrence of PPD was significantly associated with higher BMI such that a 1 unit increase in BMI was associated with a 9% increase in the odds of PPD (*P* = 0.012; 95% CI: 1.01–1.16).

A trend towards increased odds of PPD with having an antepartum history of preexisting psychiatric illness was noted though the association was not statistically significant (*P*=0.062)

## Discussion

Peripartum depression is associated with poor infant growth and development, decreased maternal attachment to children and increased risk of harm to both the mother and the newborn. The largest predictors for PPD are anxiety and depression during the pregnancy or postpartum, lack of social support, past perinatal loss, history of mood disorder such as bipolar disorder, and stressful life events [2,8]. Our study did not find an association between utilization of labor epidural analgesia and decreased rates of PPD. Our study contrasts a previous prospective study by Ding et al, which demonstrated that labor epidural analgesia was associated with a decreased likelihood of postpartum depression [6]. Failure of our study to replicate the findings by Ding, et al., suggests that labor epidural analgesia is perhaps not an intervention which would decrease the incidence of PPD. This is unfortunate given the paucity of accessible and modifiable options for this serious clinical condition. Nevertheless, our study revealed that there are two potential areas for future research and intervention that could be risk reduction strategies for PPD. First, an association was noted between PPD and women with a higher BMI. Health care initiatives in weight management programs for women of child-bearing age would be a modifiable intervention for stemming this clinical and public health problem of PPD. The second finding was a trend towards increased odds of developing PPD in parturients with an antepartum history of psychiatric illness. Other studies have shown a history of previous psychiatric illness to be a risk factor for PPD [2,8]. Therefore, an important risk reduction strategy for PPD would include antepartum screening in parturients to identify modifiable risk factors for PPD.

Our findings are consistent with prior studies demonstrating that postpartum depression affects approximately 20% of women.^1^ These findings underscore the recent United States Preventative Services Task Force guidelines to screen all pregnant and postpartum women for peripartum depression. The limitations of our study include a small sample size and assessment of depression by EDPS questionnaire based on self-reporting rather than a formal diagnostic interview.

While utilization of labor epidural analgesia would have been an attractive preventable strategy for development of PPD, our study was unable to demonstrate it. Additional studies are needed to further evaluate these findings and identify other possible preventative interventions for the reduction of peripartum depression.

## Figures and Tables

**Table 1:** Patient characteristics by PPD status. Continuous variables are reported as mean (SE) and categorical variables are reported as n (%).

	No PPD (n=52)	PPD (n=13)	*P*
Age (years)	28.4 (0.79)	27.1 (1.67)	0.490
BMI (kg/cm^2^)	27.3 (1.08)	33.9 (2.68)	0.012
Obese	15 (28.9)	7 (53.9)	0.109
Gravida	2.6 (0.24)	2.8 (0.46)	0.717
Gestational Age at Birth	38.3 (0.37)	38.5 (0.32)	0.720
Epidural (Yes)	38 (73.1)	12 (2.3)	0.268
Parity			
0	20 (38.5)	7 (53.9)	0.598
1	23 (44.2)	4 (30.8)	
2	5 (9.62)	2 (15.4)	
3	4 (7.96)	0 (0.00)	
Race			
White	34 (65.4)	7 (53.9)	0.596
African American	15 (28.8)	5 (38.5)	
Other	3 (5.77)	1 (7.69)	
Relationship with Father (Yes)	49 (96.1)	12 (92.3)	0.500
Education Level (High School or Less)	16 (31.4)	5 (38.5)	0.343
Income (<$12,000)	7 (14.9)	5 (38.5)	0.110
History of Psychiatric Illness	16 (32.0)	8 (61.5)	0.062
